# Identification of the Exercise and Time Effects on Human Skeletal Muscle through Bioinformatics Methods

**DOI:** 10.1155/2022/9582363

**Published:** 2022-08-21

**Authors:** Mufang Feng, Jie Ji, Xiaoliu Li, Xinming Ye

**Affiliations:** ^1^School of Sports Science and Engineering, East China University of Science and Technology, 130 Meilong Road, Shanghai 200237, China; ^2^Department of Rehabilitation, Minhang Hospital Fudan University, 170 Xin-Song Road, Shanghai 201199, China

## Abstract

**Background:**

The human body has more than 600 kinds of skeletal muscles, which accounts for about 40% of the whole weight. Most skeletal muscles can make bones move, and their strength and endurance directly affect their performance during exercise.

**Methods:**

To determine the effects of exercise and time on human skeletal muscle, we downloaded the microarray expression profile of GSE1832 and analyzed it to select differentially expressed genes (DEGs). Then, a protein-protein interaction (PPI) network was established, and the hub genes were identified. Afterwards, DEGs were applied to perform Gene Ontology (GO) and Kyoto Encyclopedia of Genes and Genomes (KEGG) enrichment analysis. Finally, with the help of Gene Set Enrichment Analysis (GSEA), the gene sets in the 7 samples were enriched in the KEGG pathway.

**Results:**

Through a series of bioinformatics analyses, we obtained a total of 271 DEGs. After that, four hub genes were determined through the PPI network, namely, EP300, STAT1, CDKN1A, and RAC2. In addition, we got that these DEGs were enriched in GO, such as regulation of cell population proliferation, cellular water homeostasis, and so on, and in KEGG, namely, hepatitis B, Epstein–Barr virus infection, small cell lung cancer, pathways in cancer, and others. Finally, the gene set in the samples obtained by GSEA was enriched in the cell cycle, chemokine signaling pathway, DNA replication, cytokine receptor interaction, ECM receptor interaction, and focal adhesion in KEGG.

**Conclusion:**

The findings obtained in this study will provide new clues for elucidating the mechanism of exercise and time on human skeletal muscles.

## 1. Background

Exercise is a variety of activities that human beings gradually develop in the cultivation process, covering various forms, such as walking, running, jumping, shooting, and dancing [1]. According to the question whether the human body has sufficient oxygen supply during exercise, it is classified into aerobic exercise and anaerobic exercise [2]. Aerobic exercise occurs when the amount of oxygen inhaled by the human body equals the amount of oxygen required, resulting in physiological balance [3]. Anaerobic exercise is defined as activity performed by human muscles, while their energy supply and metabolism are anaerobic [4]. Many studies have demonstrated that exercise is related to reducing the risk of death and preventing cancer diseases [5]. In addition, it also functions importantly in the treatment of obesity, cardiovascular disease, chronic obstructive pulmonary disease, diabetes, osteoporosis, osteoarthritis, and so on [6, 7]. Although it is currently known that exercise can improve human health and prevent diseases, the specific molecular mechanism of its action is still unclear.

Bioinformatics integrates biology, computer science, mathematics, physics, and so on, as an interdisciplinary subject with great development potential [8]. After data from biological experiments is acquired, processed, stored, retrieved, and analyzed, the aim of revealing the biological significance of the data and interpreting the laws of life activities has been achieved [9]. At present, bioinformatics technology mainly processes various data of proteome, which is also an important content of proteome research [10]. The microarray, also called the oligonucleotide array, is a new technology developed based on the original nucleic acid hybridization by integrating microelectronics, life science, computer science, and photoelectrochemistry [11]. Masotti, et al. believed that microarray technology could be used to comprehensively evaluate gene expression profiles and understand the factors that control gene transcription regulation and has broad application prospects in nutrigenomics research [12]. To study the pathogenesis of breast cancer, Cooper et al. analyzed the classification of breast cancer, disease development, DNA sequencing, mutation detection, and tumorigenesis through microarray technology [13]. At present, this technology is widely used to search for the expressions of various specific genes and diseases, which is of great significance for exploring the mysteries of human diseases and revealing the nature of diseases.

Herein, we comprehensively used bioinformatics to study the effects of exercise and time on human skeletal muscles. First, we downloaded the GSE1832 microarray data set from the Gene Expression Omnibus (GEO) database and performed differentially expressed genes (DEGs) analysis on 7 samples. Then, the functions and signal pathways of DEGs were enriched and analyzed. After that, the protein-protein interaction (PPI) network was established to determine the hub genes and analyzed their biological function. Gene Ontology (GO) and Kyoto Encyclopedia of Genes and Genomes (KEGG) and Gene Set Enrichment Analysis (GSEA) were also performed. The above research might provide a basis for elucidating the effects of exercise and time on human health.

## 2. Materials and Methods

### 2.1. Data Processing

In the GEO database (https://www.ncbi.nlm.nih.gov/geo/), the gene expression profile of GSE1832 was firstly submitted in 2004, containing 15 samples [14]. Since this study mainly discusses the effects of exercise and time on human skeletal muscles, only 4 samples 6 hours after exercise were selected as the control group and 3 samples 18 hours after exercise as the case group for the next step of the study. Exercise causes structural changes in muscle and can induce phase shifts in circadian rhythms. Muscle biopsies were performed 6 hours (between 1930 and 2000 hours) and 18 hours (between 0730 and 0800 hours the next day) after resistance exercise in the exercised leg. Therefore, the two groups we choose can not only explore the effects of exercise and time on human bones but also study the effects of exercise on circadian rhythm.

### 2.2. Identification of DEGs

To obtain DEGs from the two sets of samples, we used the limma package in *R* software to analyze and compare 7 samples, set fold change (FC) > 1, adjusted the *P* value *<*0.05 for DEGs screening, and filtered out the remaining DEGs [15, 16].

### 2.3. PPI Construction and Hub Gene Selection

We uploaded the information obtained from DEGs to the Search Tool for the Retrieval of Interacting Genes (STRING, https://string-db.org/) database to construct the PPI network and exported the TXV format and then used Cytoscape software to adjust and beautify the network [17]. The nodes in the PPI network represented proteins, and the lines represented the interactions between proteins [18]. Finally, the hub genes were screened out based on the gene degree in the network, and their expressions in the two sets of samples were compared to verify their biological effects.

### 2.4. Enrichment Analysis of DEGs

In this study, we analyzed the enrichment of DEGs in GO function and the KEGG pathway through the Database for Annotation, Visualization and Integrated Discovery (DAVID) database. Among them, the DAVID database is an online analysis software that can be used for gene differential analysis and pathway enrichment. GO is widely applied in bioinformatics analysis, and cell component (CC), molecular function (MF), and biological process (BP) are its three elements [19]. KEGG is a database developed by the Supercomputer Laboratory of the Institute of Chemistry, Kyoto University, Japan, in 1995. It includes pathways used to represent higher-order functions of interacting molecular networks, GENES used to collect gene catalogs of all fully sequenced genomes and some partial genomes, and LIGAND used for chemical collection [20].

### 2.5. GSEA Analysis

GSEA is a technique that uses fundamental knowledge to disclose genomic expression data. It sorts genes based on the expression degree in the two types of samples according to the predefined gene set according to FC [21]. Here, we performed enrichment analysis on the gene set in the sample in the KEGG pathway based on GSEA. *P* < 0.05 indicated statistical significance.

## 3. Results

### 3.1. Identification of DEGs

**Figure 1:**
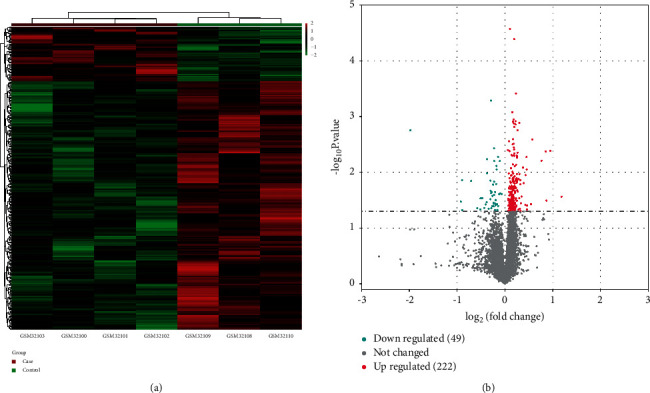
Differential analysis of DEGs. (a) Heat map of DEGs. The red part represents the case group, which is a sample of 3 cases 18 hours after exercise. The green part represents the control group, which is a sample of 6 hours after 4 exercises. (b) Volcano map of DEGs. Blue dots represent the downregulated DEGs, and red dots represent the upregulated DEGs.

Under the set DEG screening conditions, after analyzing the information in the two sets of samples through the limma package of the *R* language, we obtained a total of 271 DEGs. The heat map exhibited the cluster expression of these DEGs in 7 samples (Figure 1(a)). The distribution of DEGs was displayed in volcanic maps (Figure 1(b)).

### 3.2. PPI Network and Hub Gene Expression Analysis

**Figure 2:**
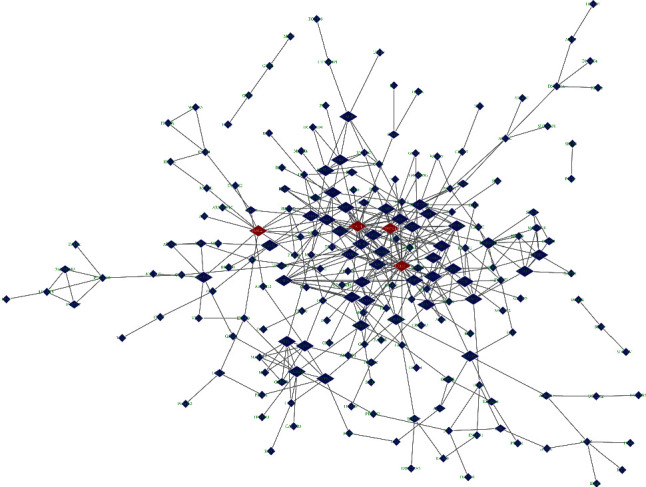
PPI network. The diamond-shaped nodes represent genes, the edges between nodes represent the degree of interconnection between genes, and the hub genes are identified by red nodes.

**Figure 3:**
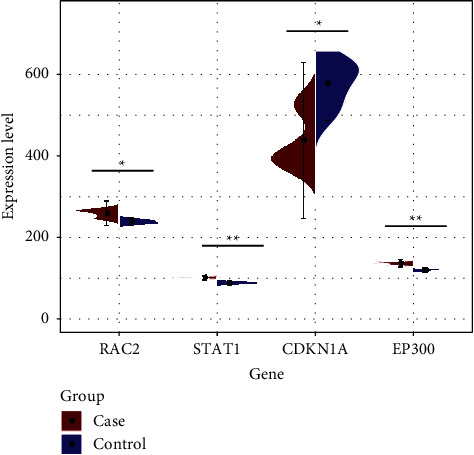
Analysis of hub gene expression in two sets of samples. The horizontal axis is 4 hub genes, and the vertical axis is their expression levels in the case group and the control group. ^*∗*^*P* < 0.05; ^*∗∗*^*P* < 0.01.

The PPI network was established based on the STRING database and Cytoscape software. It could be seen from Figure 2 that the network was composed of 206 nodes and 399 edges. On this basis, we sorted the degree size of all genes in the figure and finally selected the genes with the degree value in the top 4 as the hub genes in this study. They were EP300 (degree= 29), STAT1 (degree= 22), CDKN1A (degree= 16), and RAC2 (degree= 12). Immediately after, we compared the expression of these 4 hub genes in the two sets of samples and found that RAC2, STAT1, and EP300 were highly expressed in the case group, while CDKN1A was relatively highly expressed in the control group (Figure 3).

### 3.3. GO Function and KEGG Pathway Enrichment Analysis of DEGs

**Figure 4:**
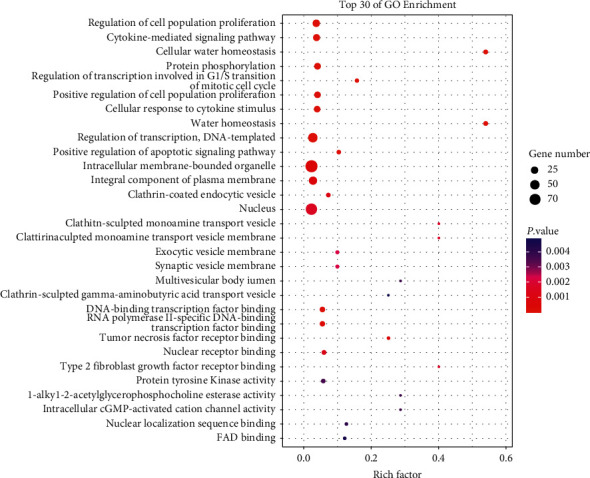
Scatter plot of GO function enrichment results. The top 30 functions of DEG enrichment in GO.

**Figure 5:**
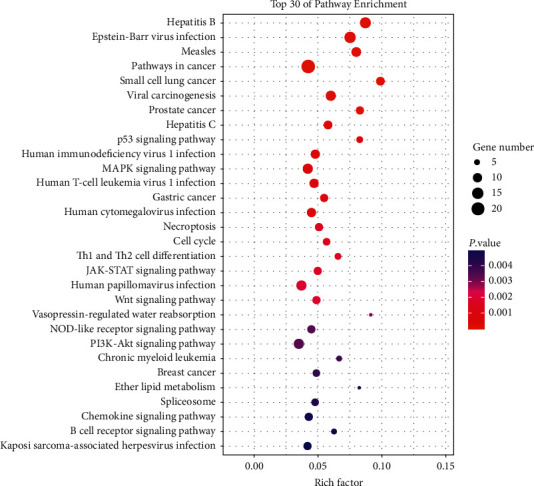
Scatter plot of KEGG pathway enrichment results. The top 30 KEGG enrichment pathways of DEGs.

We uploaded the information of 271 DEGs to the DAVID database. Finally, the first 30 functions enriched by DEGs in GO were screened, namely, regulation of cell population proliferation and transcription, DNA-templated, nucleus, cytokine-mediated signaling pathway, cellular water homeostasis, protein phosphorylation, intracellular membrane-bounded organelle, and so on (Figure 4). Next, Figure 5 also displays the top 30 KEGG pathways enriched by DEGs, including, Epstein–Barr virus infection, hepatitis B, measles, pathways in cancer, small cell lung cancer, viral carcinogenesis, prostate cancer, hepatitis C, p53 and MAPK signaling pathways, and so on.

### 3.4. GSEA Analysis

**Figure 6:**
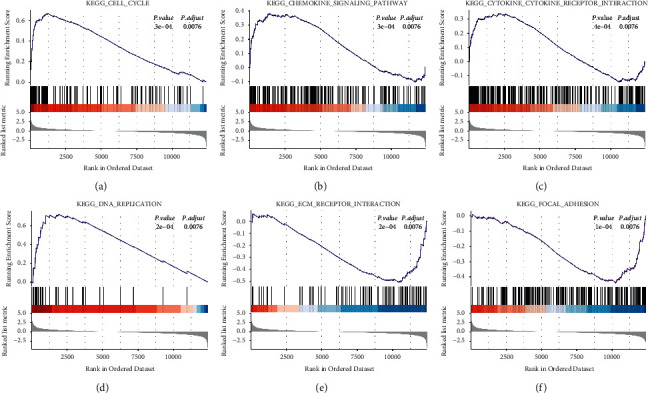
GSEA enrichment analysis of the gene set in the KEGG pathway. The gene profiles of samples were significantly enriched in the (a) cell cycle, (b) chemokine signaling pathway, (c) cytokine receptor interaction, (d) DNA replication, (e) ECM receptor interaction, and (f) focal adhesion. GSEA, gene set enrichment analysis; KEGG, Kyoto Encyclopedia of Genes and Genomes.

GSEA was used to analyze the biological function and key pathway enrichment of the gene set in the samples. At the end of the research, to explore the potential functions of the poplar root gene set, we analyzed the gene set in the sample in KEGG based on GSEA. As a result, the enriched pathways were obtained, namely, cell cycle (NES = 2.77, *P*=0.0003), chemokine signaling pathway (NES = 1.68, *P*=0.0003), DNA replication (NES = 2.36, *P*=0.0002), cytokine receptor interaction (NES = 1.55, *P*=0.0004), ECM receptor interaction (NES = −1.85, *P*=0.0002), and focal adhesion (NES = −1.80, *P*=0.0001, Figure 6).

## 4. Discussion

Regular exercise helps to curb the occurrence and development of human diseases [22]. Levine, et al. defined the thermogenesis of nonexercise activities [23]. All physical activity energy expenditure except for structural exercise has been found to have certain functions in humans' resistance to fat gain. Daily physical activity at home and in the workplace may be a kind of mild to moderate exercise intensity related to reducing the risk of many chronic diseases [24]. Yuan et al. believed that regular aerobic activity over a lengthy period can help to decrease arterial stiffness and enhance arterial anatomy [25]. On the contrary, lack of exercise is considered to be an important reason for the increase in cardiovascular disease morbidity and mortality. In addition, bioinformatics analysis provides reliable data support for elucidating the mechanism of regular exercise regulating human health.

In this study, we studied the effects on skeletal muscles 6 hours after exercise and 18 hours after exercise. We performed GO and KEGG enrichment analysis on DEGs and obtained the first 30 key pathways. Among them, in GO enrichment analysis, the cytokine-mediated signaling pathway is related to skeletal muscle growth. For example, the study by Stephanie, et al. showed that the related interference of endoplasmic reticulum stress and calcium signal was related to cytokine-mediated skeletal muscle and function [26]. Seok Won Chung's research showed that aquaporin 4 (AQP 4) regulated the flux of water on the cell membrane and maintained cell homeostasis. Because AQP 4 is enriched in skeletal muscle sarcolemma, the functional defect of AQP 4 may lead to skeletal muscle dysfunction [27]. Recent studies have also reported that the phosphorylation of 19S proteasome subunit Rpn6 activates 26S proteasome after high-intensity exercise in human skeletal muscle [28]. In the KEGG enrichment analysis, Vickie, et al. believed that the etiology of cancer-related muscle atrophy was multifactorial. Tumor metabolism captures energy fuel and amino acids, and a group of tumor-derived molecules triggers catabolic pathways at the muscle tissue level [29]. Endocrine, neurological, and inflammatory disorders increase further breakdown drives. Antitumor drugs have a great contribution to muscle wasting by directly acting on muscle cells and through their systemic side effects. Zolfaghari, et al. found that endurance exercise significantly modified the activity of the p53/ATF4/p21 signaling pathway [30]. The results of Ashutosh, et al. showed that LRRC8 complex regulates insulin-PI3K-AKT-mTOR signal in skeletal muscle, which affects the differentiation of skeletal muscle in vitro and the size of the skeletal myofiber [31]. These observations suggest that exercise mediates these signaling pathways to affect skeletal muscle.

A total of 4 hub genes were obtained from the PPI results, including RAC2, STAT1, CDKN1A, and EP300. RAC2 (small GTPase 2 of the RAC family) in the hub gene is a protein-coding gene. The diseases associated with RAC2 include immunodeficiency 73A with neutrophil chemotaxis and leukocytosis and immunodeficiency 73C with neutrophil chemotaxis and hypoglobulinemia [32]. Maik studies show that RAC2 is required for myotube migration through differentially regulating cell-matrix adhesion [33]. The protein encoded by STAT1 is a member of the STAT protein family. The diseases associated with STAT1 include immunodeficiency 31B and immunodeficiency 31C [34]. The STAT1 phosphorylation level was elevated significantly 2 and 6 h after exercise and then returned to baseline 24 h after exercise [35]. The study by Wang et al. showed that miR-208B participated in the cell cycle and proliferation regulation of bovine skeletal muscle cells through the posttranscriptional downregulation of CDKN1A [36]. Diseases associated with EP300 include Rubinstein–Tabby syndrome 2 and Menke–Hennekam syndrome 2 [37]. In addition, EP300 histone acetyltransferase (HAT)-mediated BDNF signaling activation might contribute to the neuroprotective effects of maternal exercise [38]. These hub genes were verified to have special expressions in skeletal muscles. We compared the expression of these 4 hub genes in the two sets of samples and found that RAC2, STAT1, and EP300 were highly expressed in the case group, while CDKN1A was relatively highly expressed in the control group. Our research has shown that RAC2, STAT1, and EP300 can act as upregulated genes, while CDKN1A can act as a downregulated gene in the samples.

In GSEA analysis, the gene set is enriched in the cell cycle, chemokine signaling pathway, DNA replication, cytokine receptor interaction, ECM receptor interaction, and focal adhesion. Miyoshi, et al. believed that the nucleus of skeletal muscle could reenter the cell cycle, suggesting that electroporation *in vivo* could induce dedifferentiation of mammalian skeletal muscle [39]. Hardy, et al. believed that the synthesis of chemokines and certain heparin sulfates has proven to be crucial in the process of muscle regeneration [40]. Chantal, et al. revealed that exercise induced additional and greater changes in gene expression in mdx mice, and the greatest effects of exercise were related to immune function or extracellular matrix (ECM) interaction [41]. Franchi, et al. found that focal adhesion kinase activity was related to contraction-dependent structural remodeling, suggesting that FAK had a potential role in directing muscle structural changes in response to different mechanical stimuli [42]. However, our study has some limitations. First of all, the potential molecular pathways of the hub genes still need to be verified by experiments. Secondly, the number of samples in the original dataset is relatively small. Therefore, it is necessary to study potential biomarkers by using larger sample sizes.

In summary, this study uses a biological information-based method to reveal DEGs related to skeletal muscle before and after exercise. Therefore, this research provides a new perspective for understanding the molecular functions of skeletal muscle before and after exercise. Nevertheless, further research is required to validate and verify the expected outcomes.

## Data Availability

The datasets used and/or analyzed during the current study are available from the corresponding author on reasonable request.
